# High-Intensity Interval Training Improves Markers of Oxidative Metabolism in Skeletal Muscle of Individuals With Obesity and Insulin Resistance

**DOI:** 10.3389/fphys.2018.01451

**Published:** 2018-10-31

**Authors:** Mariana Aguiar de Matos, Dênia Vargas Vieira, Kaio Cesar Pinhal, Jennifer Freitas Lopes, Marco Fabrício Dias-Peixoto, José Rodrigo Pauli, Flávio de Castro Magalhães, Jonathan P. Little, Etel Rocha-Vieira, Fabiano Trigueiro Amorim

**Affiliations:** ^1^Programa Multicêntrico de Pós-Graduação em Ciências Fisiológicas, Universidade Federal dos Vales do Jequitinhonha e Mucuri, Diamantina, Brazil; ^2^Laboratório de Biologia Molecular do Exercício, Faculdade de Ciências Aplicadas, Universidade Estadual de Campinas, Limeira, Brazil; ^3^School of Health and Exercise Sciences, The University of British Columbia, Vancouver, BC, Canada; ^4^Department of Health, Exercise, and Sports Sciences, University of New Mexico, Albuquerque, NM, United States

**Keywords:** insulin signaling exercise, obesity, mitogen-activated protein kinases, inflammation, skeletal muscle

## Abstract

**Background:** The excess body fat characteristic of obesity is related to various metabolic alterations, which includes insulin resistance (IR). Among the non-pharmacological measures used to improve insulin sensitivity are aerobic physical training, such as high-intensity interval training (HIIT). This study investigated the effects of 8 weeks of HIIT on blood and skeletal muscle markers related to IR and oxidative metabolism in physically inactive individuals with obesity and compared the changes between insulin resistant and non-insulin resistant phenotypes.

**Methods:** Initially to investigate the effect of obesity and IR in the analyzed parameters, insulin-sensitive eutrophic volunteers (CON; *n* = 9) and obese non-insulin (OB; *n* = 9) and insulin-resistant (OBR; *n* = 8) were enrolled. Volunteers with obesity completed 8 weeks of HIIT in a cycle ergometer. Venous blood and *vastus lateralis* muscle samples were obtained before and after the HIIT. Body composition and peak oxygen consumption (VO_2_peak) were estimated before and after HIIT.

**Results:** HIIT reduced IR assessed by the homeostatic model assessment of insulin resistance (HOMA-IR) in OBR (4.4 ± 1.4 versus 4.1 ± 2.2 μU L^−2^), but not in OB (HOMA-IR 1.8 ± 0.5 versus 2.3 ± 1.0 μU L^−2^) volunteers. HIIT increased VO_2_peak with no change in body fat in both groups. In skeletal muscle, HIIT increased the phosphorylation of IRS (Tyr612), Akt (Ser473), and increased protein content of β-HAD and COX-IV in both groups. There was a reduction in ERK1/2 phosphorylation in OBR after HIIT.

**Conclusion:** Eight weeks of HIIT increased the content of proteins related to oxidative metabolism in skeletal muscle of individuals with obesity, independent of changes total body fat.

## Introduction

The obesity epidemic is a worldwide public health problem. Recent data indicates that over 650 million adults around the world (∼13%) are considered obese (WHO, 2017). Obesity is one of the major risk factors for type 2 diabetes mellitus (T2D) (Shaw et al., 2010; Zhang et al., 2010) and 90% of T2D cases are attributable to excess weight (Hossain et al., 2007). The connection between obesity and T2D is believed to be intrinsically related to insulin resistance (IR) (Felber and Golay, 2002). IR is characterized by a blunted effect of insulin on decreasing circulating blood glucose at the whole-body level and/or a lower response of certain tissues to the action of insulin, such as skeletal muscle. In this context, skeletal muscle is responsible for ∼70–80% of insulin-stimulated post-prandial glucose uptake and plays a key role in the maintenance of whole-body insulin sensitivity and control of glycemic homeostasis (DeFronzo et al., 1981; Zierath et al., 2000). Additionally, IR in skeletal muscle is one of the earliest detectable defects preceding hyperglycemia, present up to 10 years before diabetes is diagnosed (Di Meo et al., 2017).

In obesity, skeletal muscle IR might be caused by several factors including accumulation of intramyocellular lipid and lipid intermediates, mitochondrial dysfunction (Sethi and Vidal-Puig, 2007), reduction in slow oxidative fibers (Oberbach et al., 2006), and activation of inflammatory pathways (Wu and Ballantyne, 2017). For example, an increase in the concentration of circulating fatty acids leads to excessive supply of lipids to the skeletal muscle that is not balanced by an increase in the oxidative metabolism. The excessive presence of lipids culminates in the accumulation of ectopic fat and lipid intermediates, such as ceramides, acylcarnitines, and diacylglycerol (DAG), interfering with the insulin signaling pathway (Schmitz-Peiffer et al., 1997; Itani et al., 2002; Aguer et al., 2015). Furthermore, pro-inflammatory cytokines can act directly on components of the insulin signaling pathway or activate mitogen-activated protein kinases (MAPKs), such as extracellular signal regulated kinases 1 and 2 (ERK1/2), N-terminal C-Jun kinase (JNK), and p38, which can directly inhibit insulin signaling (Hirosumi et al., 2002; Fujishiro et al., 2003; Cuenda and Rousseau, 2007). Other studies suggest that IR could be related to the impairment in oxidative capacity as consequence of both changes in fiber composition and in fiber-specific metabolism (Oberbach et al., 2006). It has been reported that glycolytic capacity is higher and oxidative capacity is reduced in skeletal muscle of individuals with type 2 diabetes (Simoneau and Kelley, 1997). Reduction of mitochondrial content due to the low expression of nuclear genes that regulate mitochondrial biogenesis, such as the co-activator of gamma-activated peroxisome 1 alpha (PGC- 1 α) and the mitochondrial transcription factor A (TFAM), resulting in insufficient oxidative capacity or mitochondrial dysfunction (Wu et al., 1999; St-Pierre et al., 2003) are observed in individuals with IR.

Although the cause of obesity-induced IR in skeletal muscle is complex and multifactorial, the beneficial effects of exercise training in preventing and treating whole-body and skeletal muscle IR are well-established (Keshel and Coker, 2015; Bird and Hawley, 2016). In individuals with IR, an improvement in insulin action on skeletal muscle glucose metabolism is one of the main effects induced by exercise training (Henriksen, 2002). Despite the efficacy of exercise training on IR, the majority of individuals do not achieve the minimum recommended levels of physical activity: 30 min or more of moderate intensity physical activity at least 5 days a week, or 20 min of vigorous physical activity at least 3 days a week, in addition to activities of daily living (Thompson et al., 2013). Among the barriers generally reported for a regular physical activity program are the lack of time, difficulty in accessing facilities for exercise, and low motivation (Sherwood and Jeffery, 2000; Korkiakangas et al., 2009).

In this context, the high-intensity interval training (HIIT), which consists of short periods of intense exercise (≥80% of maximal heart rate) alternated with periods of active recovery or rest, is a relatively time-efficient training strategy to improve metabolic health (Helgerud et al., 2007) and has been considered more enjoyable than moderate-to-vigorous intensity continuous training (Kong et al., 2016). It is worth highlighting that HIIT differs from the sprint interval training in which bouts are performed with supramaximal exercise intensities (i.e., “all-out” manner) and have a relatively high volume of exercise (Gibala, 2018). HIIT increases skeletal muscle oxidative capacity and induces physiological remodeling that are similar to the changes promoted by continuous moderate-intensity aerobic training (Gibala et al., 2014). As recently reviewed by Jelleyman et al. (2015), HIIT appears effective in improving insulin sensitivity, particularly in those at risk of, or with, T2D. A previous study (Little et al., 2011a) using 10 × 60-s cycling intervals (∼90% of maximal heart rate) interspersed with 60 s of recovery, three times per week over a period of 2 weeks, reported reduced hyperglycemia and increased skeletal muscle oxidative capacity in patients with T2D.

The molecular mechanism related to the effect of HIIT on skeletal muscle of individuals with IR and obesity has not been thoroughly investigated. It seems that the effect of HIIT on IR is partly mediated by improved skeletal muscle mitochondrial content/function (Little et al., 2011a), as HIIT has been shown to be a potent activator of upstream signals to mitochondrial biogenesis such as PGC1-α (Burgomaster et al., 2008; Gibala et al., 2009), and TFAM (Little et al., 2011b). Although prolonged moderate intensity continuous exercise training induces adaptations that increase the content of key signaling proteins related to glucose metabolism, such as the insulin receptor, protein kinase B (Akt), Akt substrate of 160 kDa (AS160) and glucose transporter 4 (GLUT4) (Little et al., 2010; Marcinko et al., 2015), the effects of HIIT in skeletal muscle of obese insulin resistant individuals remain unclear. To date, no study has compared the effects of HIIT on skeletal muscle markers of oxidative capacity, mitochondrial biogenesis, and insulin signaling of insulin resistant and non-insulin resistant individuals with obesity. The changes induced by the HIIT training might be blunted in individuals with IR.

Therefore, the current study was designed to investigate the effects of HIIT on blood and skeletal muscle markers related to IR, MAPKs, mitochondrial biogenesis and oxidative metabolism in physically inactive insulin resistant and non-insulin resistant individuals with obesity. We hypothesized that 8 weeks of HIIT would improve insulin sensitivity in both groups, but it would be greater in obese individuals who were insulin resistant. Additionally, we hypothesized that HIIT would be more effective in improving markers of insulin signaling, MAPKs, mitochondrial biogenesis, and oxidative metabolism proteins in the skeletal muscle of insulin resistant individuals with obesity. A group of insulin sensitive non-obese individuals was recruited for the baseline measurements. These subjects did not participate of the 8 weeks of HIIT and their results were used to differentiate a non-insulin resistant obese to a normal weight insulin resistant individual.

## Materials and Methods

### Participant Recruitment

This study was approved by a local Research Ethics Committee (protocol # 054/10) and registered in the Brazilian Registry of Clinical Trials (RBR- 6g3ngr^1^). Before agreeing to participate in the study, the participants gave their written informed consent.

Participants were recruited from the local community through poster advertisement and one-to-one interaction. An initial screening interview was used to select individuals with body mass index (BMI) within 18.5 and 24.9 kg/m^2^ or ≥30 kg/m^2^. Exclusion criteria included self-report of: (1) smoking within the past 6 months; (2) current use of anti-inflammatory, hypoglycemic, or other drugs known to affect metabolism; (3) body mass change ≥ 3 kg within the past 3 months, (4) diagnoses of T2D or glucose intolerance or any acute or chronic diseases. Subjects who met the initial requirements underwent a fasting blood draw for insulin and glucose measurements and oral glucose tolerance test (OGTT). Individuals with altered glucose metabolism (fasting plasma glucose > 100 mg/dL or end point 2-h OGTT plasma glucose > 140 mg/dl) were excluded.

Eligible participants were allocated in the study groups according to the classification of BMI and presence or absence of IR as defined by the homeostatic model assessment of IR (HOMA1-IR) (Matthews et al., 1985). The HOMA-IR is a valid and reliable test to evaluate insulin sensitivity and presents a strong correlation with the hyperinsulinemic euglycemic clamp technique (Sarafidis et al., 2007). A HOMA1-IR ≥ 2.71(mmol) (μU)/l^2^ was adopted to categorize individuals as insulin resistant. Nine insulin resistant (OBR) and nine non-insulin resistant obese individuals (OB), and nine non-insulin resistant non-obese controls (CON), sedentary (exercise frequency/duration < 2 times/week, 60 min/session), males and females, were selected to participate in the present study. Groups were matched by age and sex.

### Pre- and Post-exercise Training Intervention Tests

Prior to exercise training, obese participants underwent: (1) a fasting blood draw for lipid blood profile measurements; (2) anthropometrics and body composition analysis; (3) a 12-lead ECG maximal graded exercise test in a cycle ergometer supervised by a cardiologist; (4) a skeletal muscle biopsy; (5) analysis of total food intake and composition. The lean non-insulin resistant subjects underwent all testing above, except the analysis of total food intake and composition and 12-lead ECG maximal graded exercise test in a cycle ergometer supervised by a cardiologist.

After the exercise training, obese individuals repeated the testing sequence indicated above. Resting muscle biopsy and blood samples were obtained 72 h before and after the training program.

### High-Intensity Interval Training Protocol

The protocol consisted of 8 weeks of high-intensity interval exercise using electromagnetic cycle ergometer with a frequency of three times a week, totaling 24 training sessions (adapted from (Little et al., 2010). Before and after each HIIT session, the volunteer cycled for 2 min at 30 W (warm-up and cool down period). Each HIIT session consisted of 8–12 cycling exercise bouts with intensity between 80 and 110% of the peak power for 60 s followed by active recovery of 60 s at 30 W. Every HIIT session lasted between 16 and 24 min. The number of cycling bouts (8–12) and exercise intensity (80–110% of the peak power) were increased progressively and similarly between groups (Table 1). During every HIIT session, heart rate and rating of perceived exertion were measured at the end of each bout and after active recovery. All HIIT sessions were supervised by a graduate exercise physiology student or an undergrad physical therapy student.

**Table 1:** High-intensity interval training (HIIT) protocol employed on the study.

Weeks	Intensity (% maximum power)	Number of bouts	Bout duration (s)	Session duration (min)
1	80	8	60	16
2	85	8	60	16
3	90	9	60	18
4	100	9	60	18
5	100	9	60	18
6	100	10	60	20
7	110	11	60	22
8	110	12	60	24

### Measurements

#### Blood Lipid, Insulin, and Glucose Measurement

After at least 12-h fast, venous blood samples were collected for measurement of blood glucose, insulin, total cholesterol and fractions (LDL, HDL, and VLDL) and triglycerides. All the biochemical analyzes were performed by a local clinical analysis laboratory. IR [HOMA1-IR = glucose (mmol) × insulin (μU/ml) ÷ 22.5] and secretory function of pancreatic beta cells [HOMA-β = 20 × insulin (μU/ml) ÷ (glucose (mmol) − 3.5] were estimated from the fasting glucose and insulin concentrations (Matthews et al., 1985). The volunteers also completed an OGTT. After fasting blood collection, the volunteer ingested 75 g of dextrose and blood samples were taken every 30 min for the subsequent 2 h for determination of glucose and insulin concentrations. The values of glycemia and insulinemia were obtained in five timepoints: 0, 30, 60, 90, and 120 min, from which a curve was drawn and the net area under the curve (AUC) for insulin and glucose was calculated. For this, the trapezoidal method was applied, using GraphPad Prism, version 6 (GraphPad Software, Inc., United States). OGTT-derived indices of skeletal muscle insulin sensitivity index (mISI) was calculated using the rate of change in plasma glucose from its peak to its nadir (dG/dt) divided by the mean plasma insulin concentration (I) during time 30–120 min of the OGTT (mISI = dG/dt ÷ I) (Abdul-Ghani et al., 2007).

#### Anthropometric and Body Composition Measurements

Height and body mass were measured using an analog scale (Welmy, model 110, 0.1 kg precision) with coupled stadiometer (0.5 cm precision). BMI was calculated using height in m and body mass in kg. The measure of waist circumference (WC) was also performed. For this, the volunteers were instructed to stand, relax the abdomen, extend the arms along the body and separate their feet by 25 to 30 cm. The tape was passed levelly around the waist at the point between the lowest rib and the iliac crests, and the measurement was performed. Total fat mass, fat-free mass and visceral fat mass were measured by dual X-ray absorptiometry (DXA, GE Lunar, Madison, WI, United States).

#### Maximal Oxygen Consumption

Due to technical issues with the metabolic cart (K4b2, Cosmed, Italy), maximum oxygen consumption was not measured but estimated during a maximal ramp graded test protocol (Myers and Bellin, 2000) conducted on an electronically braked cycle ergometer (Corival, Medgraphics, United States). The workload was increased every 60 s at an individual rate, which was based on the participant’s exercise history questionnaire (Veterans Specific Activity Questionnaire), to induce fatigue within 8 to 12 min. Heart rate (HR) was recorded every minute using a HR transmitter strap (S810i series TM, Polar, United States). From this test, we obtained the peak power in watts, defined as the maximum power developed in the last minute of the protocol, and the peak oxygen consumption (VO_2_peak) was calculated (Thompson et al., 2013). We used the equation (equation 1) described below to calculate the VO_2_max on cycle ergometer:

Equation 1:

(1) $$\begin{array}{l} {\mathrm{VO}}_{2}({\mathrm{mL kg}}^{-1}\min^{-1})=1.8\times (\mathrm{work rate})/(\mathrm{body mass})\\ \;+3.5\;{\mathrm{mL kg}}^{-1}\min^{-1}+3.5\;{\mathrm{mL kg}}^{-1}\min^{-1}. \end{array}$$

Where VO_2_ is the oxygen consumption (mL kg^−1^ min^−1^), body mass in kilograms, and work rate in kilograms/meter.

At the fourth and eighth weeks of training, the test was repeated as described above. The maximal work rate recorded at the fourth week was used to adjust the intensity of the HIIT.

#### Analysis of Total Food Intake and Composition

The volunteers were instructed to not change their eating habits during the study period. In order to monitor their food intake (calories) and composition (macronutrients), a 3-day dietary log was used to track two weekdays and one weekend day, as suggested by Willet and Stampfer (Willett and Stampfer, 1986). The dietary log was recorded one week before the starting and at the 7^th^ week of HIIT. The data obtained using the logs were converted to weight (kg) or volume (ml) and analyzed using a commercial dietary analysis software (DietPro^®^, version 5.7, A.S. Systems, 2013). Mean values of energy (kcal) and macronutrients intake (proteins, lipids, and carbohydrates) were expressed pre- and post-HIIT for both groups.

#### Vastus Lateralis Biopsies

Skeletal muscle biopsies were performed in the *vastus lateralis* of the dominant leg using the Bergström technique (Bergstrom and Hultman, 1966) under local anesthesia (2% lidocaine with epinephrine). Samples were harvested by suction, immediately frozen in liquid nitrogen and stored in a −80°C until analysis. Muscle tissue harvesting occurred 72 h before or after the first and last HIIT session, respectively, as described by previous studies (Little et al., 2010; Hood et al., 2011). The biopsy procedures were performed in a fasted state (at least 12 h) in the morning. The second biopsy was performed approximately 5 cm away from the first at the same time of the day.

#### Determination of Protein Content and Phosphorylation in Skeletal Muscle

Skeletal muscle was homogenized, and protein content determined as previously described (de Matos et al., 2014). For Western blot analyses, muscle lysate (approximately 50 mg cellular protein) was separated by SDS-PAGE, electro-transferred onto polyvinylidene difluoride membranes (Millipore, Billerica, MA, United States), and probed overnight with phospho-SAPK/JNK (Thr183/Tyr185, Cell Signaling, #9251), phospho-p38 (Thr180/Tyr182, Cell Signaling, #9211), phospho-ERK1/2 (Cell Signaling, #9102), phospho-Akt (Ser473, Cell Signaling, #9271), phospho-IRS-1 (Ser612-C15H5, Life Sciences, #44816), phospho-AS160 (Thr642, Cell Signaling, #8881), GLUT4 (1F8, Cell Signaling, #2213), βHAD (Proteintech, #19828-1-AP), COX IV (3E1, Cell Signaling, #4850), TFAM (Cell Signaling, #7495), PGC1α (Calbiochem, # ST1202), and GAPDH (14C10, Cell Signaling, #2118) antibodies. Proteins were visualized by horseradish peroxidase-conjugated IgG antibodies and ECL SuperSignal (Milipore) and captured by a photo documentation system (L-Pix Chemi, Loccus Biotecnologia). The bands were analyzed using Scion Image software (Scion Corporation based on NIH Image; National Institutes of Health, Scion Corporation, Frederick, MD, United States). Glyceraldehyde-3-phosphate dehydrogenase (GAPDH) was used as normalizer for all proteins analyzed. GAPDH was not always ran in the same blot of the target protein. Ponceau stain was used to guarantee that protein transfer was similar among blots and unaffected by experimental conditions. The relative expression values are presented as arbitrary units. The data were related to the average optical density of the control group, which was considered as 100%. For some western blot analyzes, there are different sample numbers for each protein analyzed, which is explained by the small amount of sample available. However, we emphasize that all pre- and post-training analyzes were performed in pairs, that is, the same volunteer before and after training.

### Statistical Analyses

Data are presented as mean ± standard deviation (SD). Statistica software (v10.0, StatSoft, Inc.) was used for statistical analysis. The Shapiro–Wilk test was used to evaluate the normality of the data. To compare the characterization data between the groups (CON, OB, and OBR), one-way analysis of variance (One-way ANOVA) was used for data that presented normal distribution and the Kruskal–Wallis test for those data with non-normal distribution. Two-way ANOVA was used to evaluate the effect of training (factor 1 = pre and post 8 weeks of HIIT and factor 2 = OB and OBR) on the parameters studied when the data presented normal distribution and the Friedman test for the data with non-normal distribution. When the differences were significant, the tests were followed by Tukey or Newman–Keuls *post hoc*. The alpha level was fixed at *p* ≤ 0.05 for statistical significance.

## Results

### Participant Characteristics

Characteristics of CON, OB, and OBR are presented in Table 2. The mean age, height, VO_2_max, and peak power in the exercise test were not different among the groups. OB and OBR were not different for body mass, BMI, fat percentage, fat mass, and WC, but these values were higher in OB and OBR compared to CON. Individuals in the OBR had higher lean mass compared to CON. There were no differences among CON, OB, and OBR for fasting glucose, total cholesterol, HDL and LDL. The OBR had higher values for visceral adipose tissue, fasting insulin, AUC for insulin, as well as HOMA1-IR index compared to CON and OB. In addition, the AUC for glucose, glucose concentration at end of the 2-h OGTT, triglyceride and VLDL concentrations were higher in OBR compared to CON. We also observed that HOMA-β index were different among CON, OB and OBR, where OBR had higher HOMA-β compared to CON and OB, and OB had higher HOMA-β compared to the CON (Table 2).

**Table 2:** Anthropometric, physical fitness, and metabolic profile of study participants.

	CON	OB	OBR	*p*
Gender (male/female)	3/6	3/6	3/5	–
Age (years)	29.0 ± 11.0	32.0 ± 10.0	30.0 ± 11.0	0.704
Body mass (kg)	60.7 ± 6.2	92.4 ± 12.9^∗^	106.0 ± 19.6^∗^	<0.0001
Height (cm)	177.0 ± 0.1	162.0 ± 0.1	167.0 ± 0.1	0.175
BMI (kg/m^2^)	20.8 ± 1.7	35.1 ± 3.8^∗^	37.8 ± 4.6^∗^	0.0001
Body fat (%)	28.1 ± 8.6	43.1 ± 8.0^∗^	44.4 ± 8.1^∗^	0.004
Waist circumference (cm)	72.0 ± 4.0	103.0 ± 13.0^∗^	115.0 ± 15.0^∗^	<0.0001
Fat free mass (kg)	42.1 ± 8.7	50.8 ± 10.8	57.0 ± 11.6^∗^	0.024
Fat mass (kg)	16.1 ± 4.0	43.1 ± 8.0^∗^	46.1 ± 13.4^∗^	<0.0001
Visceral adipose tissue (g)	149 ± 80	1153 ± 431^∗^	1785 ± 754^∗&^	<0.0001
VO_2max_ (mL min^−1^ kg^−1^)	33.1 ± 7.2	27.2 ± 6.8	25.1 ± 6.2	0.331
Peak power (watts)	146.0 ± 52.0	170.0 ± 66.0	176.0 ± 67.0	0.577
Fasting insulin (μU/mL)	4.9 ± 2.0	8.8 ± 2.5	20.7 ± 5.9^∗&^	0.0001
Fasting glucose (mg/dL)	86.0 ± 3.0	84.0 ± 10.0	87.0 ± 8.0	0.717
HOMA1-IR (μU L^−2^)	1.0 ± 0.4	1.8 ± 0.5	4.4 ± 1.4^∗^	<0.0001
HOMAβ (μU L^−2^)	77.5 ± 32.8	199.4 ± 129.3^∗^	348.0 ± 144.7^∗&^	<0.0001
OGTT (mg/dL)	77.0 ± 24.0	87.0 ± 15.0	105.0 ± 20.0^∗^	0.025
AUC glucose (mg/dL^x^min)	1.110 ± 1.305	1.267 ± 2.269	1.386 ± 2.164^∗^	0.037
AUC insulin (mg/dl^x^min)	5.806 ± 2.298	9.696 ± 6.123	17.821 ± 8.109^∗&^	0.018
Total cholesterol (mg/dL)	189.0 ± 63.0	192.0 ± 26.0	196.0 ± 30.0	0.957
LDL-C (mg/dL)	120.0 ± 46.0	121.0 ± 27.0	124.0 ± 24.0	0.722
HDL-C (mg/dL)	52.0 ± 13.0	50.0 ± 12.0	41.0 ± 5.0	0.109
VLDL-C (mg/dL)	17.0 ± 8.0	20.0 ± 11.0	31.0 ± 11.0^∗&^	0.030
Triglycerides (mg/dL)	86.0 ± 40.0	102.0 ± 56.0	155.0 ± 58.0^∗&^	0.030

*Data are expressed as mean ± standard deviation. ^∗^ Difference compared to CON, ^&^ difference compared to OB. One-way ANOVA.*

### Effect of HIIT on Maximal Oxygen Uptake, Body Composition, and Blood Variables of Obese Individuals

High-intensity interval training increased VO_2_peak and peak power of both OB and OBR (*p* < 0.0001). Also, HIIT reduced body fat percentage in the OB (*p* = 0.001) and increased lean mass in the OBR (*p* = 0.006). A tendency to increase lean body mass in the OB was observed (*p* = 0.06). There was no effect of HIIT on body mass, BMI, WC, and fat mass (*p* > 0.05) (Table 3).

**Table 3:** Effect of 8-week HIIT on physical fitness, body composition, and blood variables of obese individuals.

	OB		OBR	
	Pre	Post	Pre	Post
VO_2_peak (mL min^−1^ kg^−1^)	27.2 ± 6.8	30.5 ± 3.4^∗^	24.8 ± 5.9	27.4 ± 6.1^∗^
Peak power (watts)	170.0 ± 66.0	198.0 ± 70.0^∗^	174.0 ± 63.0	200.0 ± 73.0^∗^
Body mass (kg)	92.4 ± 12.9	92.1 ± 13.1	106.2 ± 18.4	107.0 ± 19.6
BMI (kg/m^2^)	35.1 ± 3.8	34.9 ± 3.7	37.8 ± 4.3	38.0 ± 4.7
Body fat (%)	43.1 ± 8.0	42.3 ± 8.0^∗^	44.6 ± 7.6	44.3 ± 8.1
WC (cm)	103.0 ± 13.0	102.0 ± 12.0	115.0 ± 15.0	116.0 ± 13.0
Fat-free mass (kg)	50.8 ± 10.8	51.6 ± 11.1	56.9 ± 10.9	57.6 ± 11.6^∗^
Fat mass (kg)	38.7 ± 9.0	37.4 ± 8.5	46.4 ± 12.6	46.5 ± 13.4
Visceral adipose tissue (g)	1153 ± 431	1072 ± 430	1737 ± 720	1861 ± 872^∗^
Fasting insulin (μU/mL)	8.8 ± 2.5	10.5 ± 3.8	20.7 ± 5.9	15.2 ± 5.4^∗^
Fasting glucose (mg/dL) **^#^**	84.0 ± 10.0	89 ± 11.0	87.0 ± 8.0	93 ± 8.0
HOMA1-IR (μU L^−2^)	1.8 ± 0.5	2.3 ± 1.0	4.4 ± 1.4	3.5 ± 1.2^∗^
HOMAβ (μU L^−2^)	199 ± 129	179 ± 128	348 ± 144	189 ± 83^∗^
OGTT (mg/dL)	87.0 ± 15.0	85.0 ± 17.0	105.0 ± 20.0	105.0 ± 22.0
Net AUC glucose (mg/dL^x^min)	2.702 ± 530	2.762 ± 458	3.602 ± 566	4.706 ± 490
Net AUC insulin (mg/dl^x^min)	8.306 ± 2.561	7.364 ± 1.673	15.380 ± 3.241	13.845 ± 2.116
Muscle ISI	1.29 ± 0.62	1.19 ± 0.74	0.49 ± 0.44	0.54 ± 0.32
Total cholesterol (mg/dL) **^#^**	192 ± 26	179 ± 36	196 ± 30	181 ± 21
LDL-C (mg/dL) **^#^**	121 ± 27	103 ± 33	124 ± 24	113 ± 20
HDL-C (mg/dL)	50 ± 12	56 ± 15	41 ± 5	41 ± 10
VLDL-C (mg/dL)	20 ± 11	19 ± 6	31 ± 11	27 ± 11
Triglycerides (mg/dL)	102 ± 56	96 ± 31	155 ± 58	137 ± 56

*Data are expressed as mean ± standard deviation. ^∗^ Indicates difference between Pre- and post-training in the same group; ^#^ Indicates a main effect of HIIT intervention independent of the group.*

It was observed a significant interaction between HIIT (pre and post) and the obesity groups (OB and OBR) on blood markers. HIIT reduced blood insulin concentration (*p* = 0.004), HOMA-IR index (*p* = 0.02) and HOMA-β index in OBR (*p* = 0.01). Also, HIIT reduced total cholesterol (*p* = 0.03) and LDL (*p* = 0.03) in OB and OBR. There was no effect of HIIT on glucose concentration at fasting or end of 2-h of OGTT, glucose and insulin AUC, muscle ISI, HDL, VLDL, and triglycerides (*p* > 0.05) (Table 3).

### Effect of Hiit on Muscle Proteins

**FIGURE 1:**
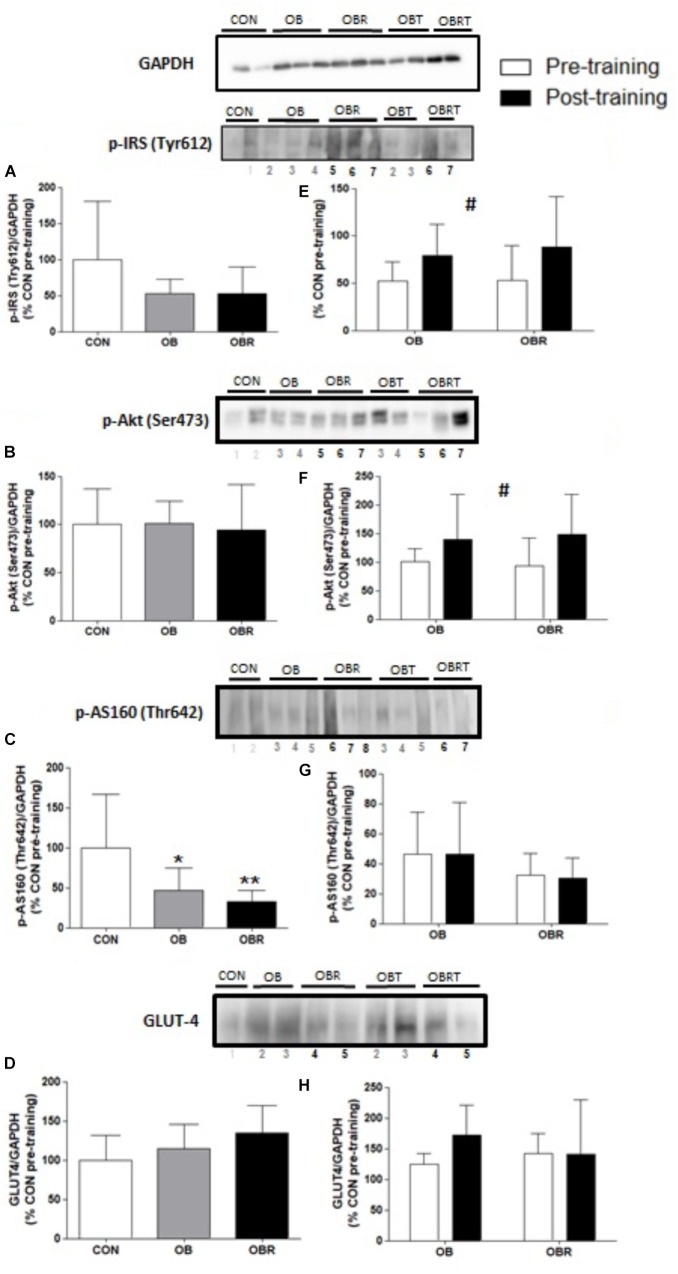
Representative blot and data of the phosphorylation of IRS (Tyr 612), Akt (Ser 473), AS160 (Thr 642) and expression of GLUT4 in skeletal muscle of CON, OB, and OBR at baseline **(A–D)** and in OB and OBR groups before and after HIIT (OBT and OBRT) **(E–H)**. Data are expressed as mean ± standard deviation, considering the control as 100%. CON: non-obese and non-insulin resistant; OB: obese non-insulin resistant pre-training; OBT: obese post-training non-insulin resistant; OBR: obese insulin resistant pre-training and OBRT: obese insulin resistant post-training. ^∗^Indicates difference between OB and CON at baseline (*p* < 0.05). ^∗∗^Indicates difference between OBR and CON at baseline (*p* < 0.05). ^#^Indicates training effect (*p* < 0.05). CON (*n* = 3–5), OB (*n* = 7–8), and OBR (*n* = 6–7).

Figure 1 shows phosphorylation and content of proteins related to the insulin signaling pathway. At baseline, phosphorylation of IRS (Tyr 612) (Figure 1B) and Akt (Ser 473) (Figure 1C), and GLUT4 content (Figure 1E) were not different among CON, OB, and OBR (*p* > 0.05). The phosphorylation of AS160 (Thr 642) was lower in the OB and OBR compared to CON (*p* = 0.03), but it was not different between OB and OBR (Figure 1D). HIIT increased IRS phosphorylation (Tyr 612) (*p* = 0.04) (Figure 1F) and Akt phosphorylation (Ser473) (*p* = 0.03) in both OB and OBR (Figure 1G). Phosphorylation of AS160 (Thr 642) (Figure 1H) and GLUT4 content (Figure 1I) were not altered by HIIT.

**FIGURE 2:**
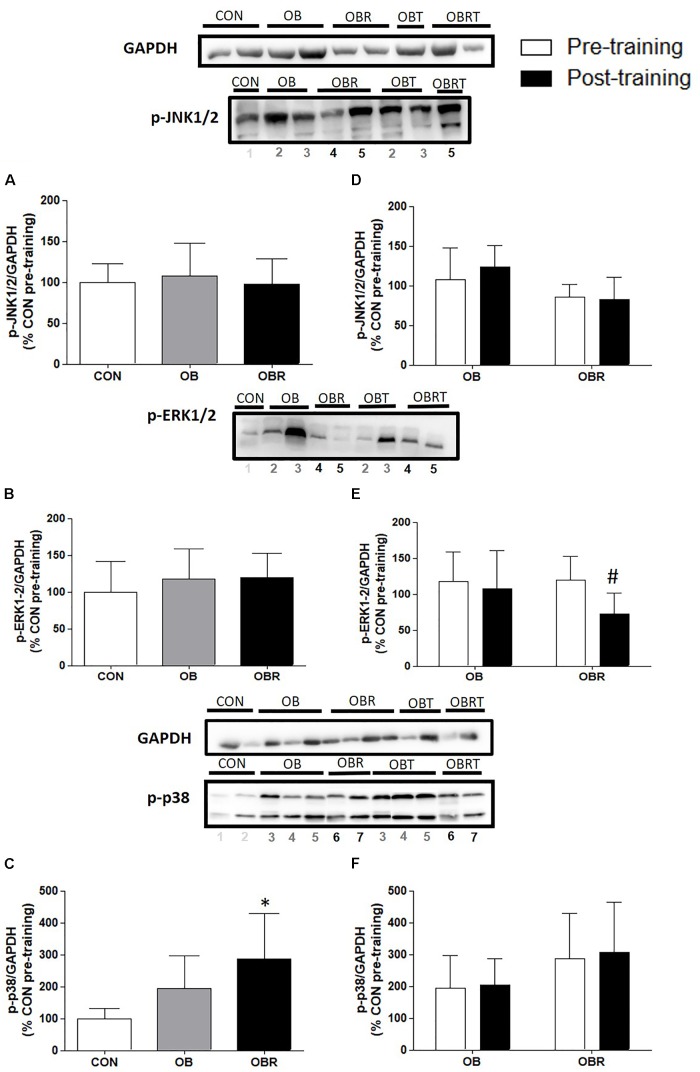
Representative blot and data of the phosphorylation of JNK1/2, ERK1/2, and p38 in skeletal muscle at baseline in CON, OB, and OBR **(A–C)** and OB and OBR groups before and after HIIT **(D–F)**. Data are expressed as mean ± standard deviation, considering the control as 100%. CON: non-obese and non-insulin resistant; OB: obese non-insulin resistant pre-training; OBT: obese post-training non-insulin resistant; OBR: obese insulin resistant pre-training and OBRT: obese insulin resistant post-training. ^∗^Indicates difference between OBR and CON in the pre-workout. ^#^Training effect. CON (*n* = 3–5), OB (*n* = 7–8), and OBR (*n* = 6–7).

In Figure 2 it is shown the proteins related to MAPK pathway in skeletal muscle. At baseline, JNK1/2 and ERK1/2 phosphorylation were not different among CON, OB and OBR, but phosphorylation of p38 in OBR was higher than CON (*p* = 0.03). After HIIT, it was observed that JNK1/2 and p38 phosphorylation did not alter, but ERK1/2 phosphorylation reduced in OBR (*p* = 0.01) (Figure 2).

**FIGURE 3:**
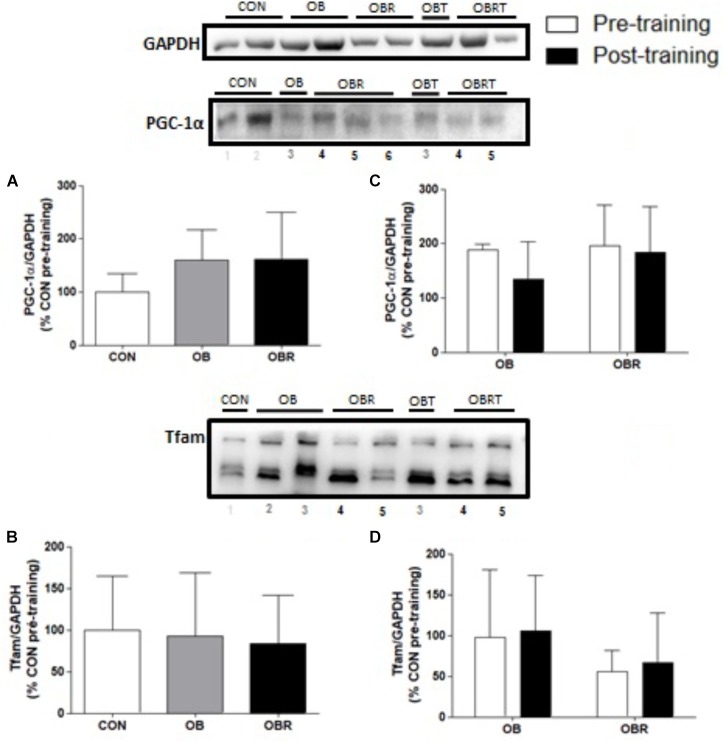
Representative blot and data of the expression of PGC-1α and TFAM in skeletal muscle at baseline in CON, OB, and OBR **(A,B)** and before and after HIIT in OB and OBR **(C,D)**. Data are expressed as mean ± standard deviation, considering the control as 100%. CON: eutrophic; CON: non-obese and non-insulin resistant; OB: obese non-insulin resistant pre-training; OBT: obese post-training non-insulin resistant; OBR: obese insulin resistant pre-training and OBRT: obese insulin resistant post-training.

Figure 3 presents the results of proteins markers related to skeletal muscle mitochondrial biogenesis and signaling pathways. At baseline content of PGC-1α and TFAM were not different among CON, OB, and OBR. After HIIT, no difference was observed for PGC-1α and TFAM on both groups (Figure 3).

**FIGURE 4:**
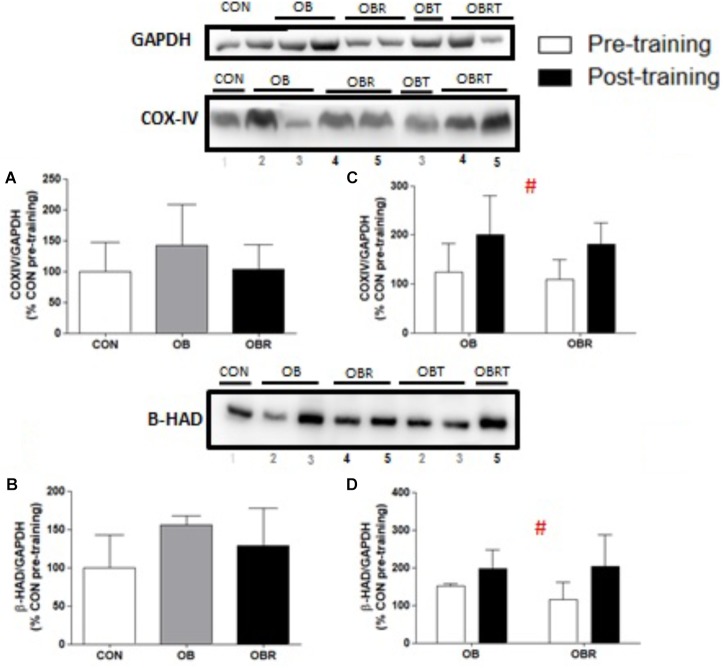
Representative blot and data of COX-IV and β-HAD expression data in the skeletal muscle in the CON, OB, and OBR groups in pre-training **(A,B)** and in the OB and OBR groups before and after HIIT **(C,D)**. Data are expressed as mean ± standard deviation, considering the control as 100%. CON: non-obese and non-insulin resistant; OB: obese non-insulin resistant pre-training; OBT: obese post-training non-insulin resistant; OBR: obese insulin resistant pre-training and OBRT: obese insulin resistant post-training. ^#^Training effect. CON (*n* = 6–7), OB (*n* = 3–4), and OBR (*n* = 5–6).

At baseline, there were no differences among CON, OB, and OBR regarding the content of both COX-IV (*p* = 0.35) (Figure 4A) and β-HAD (*p* = 0.14) (Figure 4B). After 8 weeks of HIIT, the content of these proteins increased in OB and OBR (*p* = 0.006 and *p* = 0.046, respectively) (Figures 4C,D).

### Effect of HIIT on Food Intake and Composition

When comparing energy intake and food composition before and at 8^th^ week of HIIT, it was observed a main effect for increased energy (*p* = 0.002) and carbohydrate intake (*p* = 0.004). There was a significant increase in the amount of protein intake before and at 8^th^ week of HIIT in OB and OBR (*p* = 0.002). The amount of lipids intake did not change with HIIT (*p* = 0.16) (Table 4).

**Table 4:** Food intake pattern of study participants.

	OB		OBR	
	
	Pre	Post	Pre	Post
Energy (kcal)^#^	2.820 ± 396	3.030 ± 273	2.941 ± 211	3.119 ± 154
Carbohydrates (g/day)^#^	390 ± 58	438 ± 60	423 ± 42	458 ± 33
Proteins (g/day)	135 ± 21	155 ± 20^∗^	134 ± 26	160 ± 24^∗^
Lipids (g/day)	78 ± 21	72 ± 8	79 ± 11	72 ± 10

*Data are expressed as mean ± standard deviation. ^#^Indicates a main effect of HIIT intervention independent of the group; ^∗^ Indicates difference between Pre- and post-training in the same group. OB: obese insulin sensitive (n = 8) and OBR: obese insulin resistant (n = 7). Pre: before HIIT and Post: after HIIT.*

## Discussion

The main aim of the present study was to compare the effect of HIIT on proteins involved in the insulin signaling pathway, and associated to IR such as MAPKs, mitochondrial biogenesis and oxidative metabolism in the skeletal muscle of insulin-resistant and non-insulin-resistant obese individuals. This is the first study to comprehensively compare these parameters in these populations. This study also characterized the differences among matched non-obese and non-insulin resistant controls (CON), obese non-insulin resistant (OB) and obese insulin-resistant (OBR) individuals at baseline, evaluating similar components mentioned above. At baseline, the main findings were: (1) OBR individuals had higher p38 and lower AS-160 phosphorylation than CON; and (2) OB individuals had lower AS-160 phosphorylation than CON. After 8 weeks of HIIT, the main findings were: (1) blood insulin, HOMA1-IR and HOMA-β reduced only in OBR; (2) increased IRS and Akt phosphorylation in OB and OBR; and (3) increased markers of oxidative metabolism in OB and OBR.

At baseline, it was observed lower phosphorylation of AS160 (Thr642) in OB and OBR compared to CON. AS160 is regulated by the phosphorylation of various serine and threonine residues in response to insulin and muscle contraction, and at least 10 phosphorylation sites have been identified (Kramer et al., 2006; Sakamoto and Holman, 2008). Among all these residues, Thr642 and Ser588 appear to be the key residues in the regulation of Akt-mediated GLUT4 translocation in response to insulin (Sano et al., 2003). It was observed lower phosphorylation of AS160 (Thr642) in both OB and OBR, without differences between these groups. It is proposed that even in the absence of clinical diagnosis of IR in OB, the presence of obesity could alter the insulin signaling pathway as measured by AS160 phosphorylation. For example, the mean value for HOMA-β was significant higher in OB compared to CON (almost two times higher). Although OB and OBR had similar AS160 phosphorylation but different HOMA-β and HOMA-IR, we speculate that the impairment in the insulin pathway may be upstream from AS160.

It was hypothesized that after 8 weeks of HIIT, IR would reduce in both obese groups, but the improvement would be greater in OBR. It was observed a significant reduction in fasting blood insulin concentration and IR measured by HOMA1-IR only in OBR. Although it was not observed a significant reduction in fasting blood insulin concentration in the OB group, HOMA1-IR was lower in six of nine volunteers. It is important to consider that other factors may be associated with non-responsiveness of some volunteers to HIIT such as VO_2_max, adiposity, sex and age. However, when comparing the pre-training OB and OBR groups there were no differences for these variables. Also, it was not observed a significant increase in muscle ISI with HIIT. However, the changes in muscle ISI (data not shown) were higher in OBR than OB suggesting that HIIT may affect insulin sensitivity in skeletal muscle of OBR individuals (*p* = 0.047). Previous studies have presented mixed results evaluating the effects of HIIT on IR. Several studies with obese individuals have used HIIT as a training program and demonstrated improvement in IR (Whyte et al., 2010; Hood et al., 2011). However, other studies were not successful in improving IR using HIIT. For example, recently Arad et al. (2015) reported that 14 weeks of HIIT did not improve insulin sensitivity in overweight/obese individuals. The difference in the results between the studies might be related to the exercise protocol and the subject’s physical characteristics. Arad et al. (2015) used a HIIT protocol composed of four intervals varying from 30 to 60 s at 75–90% heart rate reserve (HRR) interspersed with recovery of intervals of 180–210 s at 50% HRR and 11 min of warmup and cooldown. It means that the total duration of intense exercise varied from 2 to 4 min and 75 to 90% HRR. Our protocol had more prolonged (10 to 12 min per session of high intensity exercise) and possibly more intense (80–110% peak power) bouts of exercise, which might have induced higher skeletal muscle metabolic stress. Our results reinforce that HIIT is an effective exercise modality to reduce IR in individuals with obesity and IR. Interestingly, this adaptation occurred independently of changes in the amount of body fat, which indicates that HIIT alters other factors (i.e., insulin signaling) not associated directly with the amount of adipose tissue. Also, HIIT may have improved the oxygen supply and nutrients to the adipose tissue and improved inflammation (Aldiss et al., 2018).

After reporting that HIIT reduced IR in insulin-resistant obese individuals, we investigated whether this effect could be related to changes in proteins involved in the insulin signaling pathway in the skeletal muscle. After HIIT, it was observed higher phosphorylation of IRS (Tyr612) and Akt (Ser473) in OB and OBR, but no change in GLUT4 content and AS160 (Thr642) phosphorylation. Similar to our results, Marcinko et al. (2015) studied rats and observed that 6 weeks of HIIT promoted greater Akt phosphorylation (Ser473 and Thr308) in the skeletal muscle, although there was no change in AS160 (Thr642). It is noteworthy that although phosphorylation of AS160 (Thr642) is recognized as an important regulator of increased insulin-stimulated glucose uptake, other residues are also involved in this process. In the study by Vind et al. (2011) for example, it was reported that 10 weeks of training exercises abolished the defects in insulin-mediated AS160/TBC1D4 phosphorylation in Ser318, Ser588 and Ser751 in patients with T2D, but not Thr642. Thus, the importance of a more detailed investigation of the role of HIIT in the different residues of AS160 is emphasized. Additionally, a study found that 16 weeks of HIIT in T2D patients induced higher membrane-bound GLUT-4 and GLUT-4 mRNA levels, but no rise in overall GLUT-4 protein content (Karstoft et al., 2014). There is evidence that increased GLUT4 translocation, rather than expression, mediates enhanced post-exercise insulin action (Hansen et al., 1998). Thus, although in the present study the content of GLUT4 has not been altered by HIIT, it cannot be ruled out that translocation and consequent action of this protein has been improved. It would be informative in future studies to examine insulin signaling and GLUT4 translocation in both the basal and insulin-stimulated state following HIIT.

This study further investigated the effects of 8 weeks of HIIT on MAPKs related to IR. Previous *in vivo* and *in vitro* studies reported an association between different MAPKs and IR (Carlson and Rondinone, 2005; Jheng et al., 2012; Boyd et al., 2013). Although MAPKs are necessary for normal physiological processes, inappropriate signaling may contribute to the development of metabolic disorders (Gehart et al., 2010). At baseline, no difference in the phosphorylation of JNK1/2 and ERK1/2 was observed among CON, OB and OBR. However, it was reported higher phosphorylation of p38 in OBR. Previous studies have shown that inhibiting p38 phosphorylation improved the insulin signaling pathway. de Alvaro et al. (2004)reported that pretreatment with inhibitors of p38 MAPK restored insulin signaling and normalized insulin-induced glucose uptake in the presence of TNF-α. Furthermore, TNF-α induced serine phosphorylation of IR and IRS-1, and these effects were completely precluded by pretreatment with inhibitors of p38 MAPK. Our findings therefore support that p38 appears to be related to IR in skeletal muscle of obese individuals, making it an interesting target for future investigations. HIIT did not change JNK1/2 and p38 phosphorylation, but reduced ERK1/2 phosphorylation in the OBR group. Inhibition of the ERK signaling pathway is related to increased Akt phosphorylation and reversion of IR induced by endoplasmic reticulum stress in L6 isolated myotubes (Hwang et al., 2013). Thus, the improvements in insulin sensitivity with HIIT in the OBR may be related to the lower phosphorylation of ERK1/2.

Another possible mechanism that links obesity to IR is reduced mitochondrial content and/or oxidative metabolism dysfunction in skeletal muscle. At baseline, no differences were observed for the content of PGC1-α and TFAM among CON, OB and OBR. As reviewed by Hafizi Abu Bakar et al. (2015), the role of mitochondrial dysfunction in the development of IR is still a matter of debate. Heilbronn et al. (2007) reported that insulin resistant obese men had reduced markers of mitochondrial metabolism, including reduced expression of *PGC1*α and *COX1 mRNA*, reduced protein levels of subunits of the respiratory chain, and reduced citrate synthase activity at baseline compared with non-insulin resistant obese men that had similar body fat percentage and fitness. Other studies comparing obese individuals with non-obese individuals have reported that obesity did not alter the protein content in the skeletal muscle of PGC-1α and PGC-1β, PPARα or TFAM (Holloway et al., 2008, 2009). Although the content of some mitochondrial proteins investigated was not different between groups, we do not know if the mitochondrial function is preserved in obese patients who are resistant or not to insulin. We speculate that if a greater supply of lipids in the skeletal muscle is not balanced by an increase in oxidative metabolism, this could lead to the accumulation of lipid metabolites and the generation of oxidative stress that interfere with the insulin signaling (Itani et al., 2002).

Activation of the AMPK, ROS, and Ca^2+^ signaling molecules are required for the regulation of PGC-1α gene expression induced by the contractile activity, regulated in part through the p38 and CaMKII pathways (Zhang et al., 2014). After 8 weeks of HIIT, it was observed higher content of COX-IV and β-HAD, but no changes in PGC-1α or TFAM content in OB and OBR. It is well-established that CaMKII in together with AMPK, p38 and ROS are some of the stimuli for induction of PGC-1α and TFAM. Granata et al. (2016) reported that healthy men who underwent 4 weeks of HIIT showed no change in the protein content of PGC-1α and TFAM in skeletal muscle. In the study by Little et al. (2010) it was observed that 2 weeks of HIIT promoted in healthy men increased activity of citrate synthase, and the content of subunits II and IV of cytochrome C oxidase and TFAM. Nuclear content of PGC-1α was increased, but without change in total content. The findings of that study emphasize the importance of considering differences in the measurement of nuclear or total PCC-1α. According to some authors, the total protein content of PGC-1α may not be fully indicative of its activation, since, the activity of PGC-1α is mainly determined by its subcellular location (Wright et al., 2007), and a number of post-translational modifications (Jager et al., 2007; Canto and Auwerx, 2009).

A previous study has shown that muscle oxidative capacity is a significant predictor of insulin sensitivity (Bruce et al., 2003). Our data demonstrate that HIIT promoted increased expression of β-HAD and COX-IV, possibly indicating an improvement in oxidative metabolism and, perhaps, this may have contributed to the improvement of insulin signaling.

In this context, it is important to note that increased incomplete fatty acid oxidation can contribute to the development of IR. As revised by Muoio and Neufer (2012), the increasing flux through β-oxidation without a corresponding boost in energy demand imposes unwarranted reducing pressure on the respiratory system, which in turn impinges upon redox balance and insulin action. Conversely, if flux through βoxidation increases as a consequence of energy demand, the system then operates in metabolic balance and insulin sensitivity is maintained. Still exists the hypothesis that electron transport chain (ETC) supercomplex assembly may be an important underlying mechanism of muscle mitochondrial dysfunction in type 2 diabetes mellitus. Antoun et al. (2015) supported this by observing that the muscle of diabetic patients revealed a striking decrease in complex I, III, and IV containing ETC supercomplexes.

The present study presented some limitations that should be considered. When we measured the phosphorylation of IRS (Tyr612) and Akt (Ser 473) in skeletal muscle, we observed no difference among CON, OB, and OBR groups. It is important to highlight that we measured the phosphorylation of the components of the insulin signaling pathway without the stimulatory effect of this hormone and, therefore, we do not know whether the response would be different under insulin-stimulated conditions in the different groups evaluated. Furthermore, when measuring phosphorylated proteins without insulin stimulation, we cannot affirm that there was an improvement in the insulin signaling pathway. Another limitation of the present study is the high background present in the images of the phosphorylated proteins and that the loading controls were not ran on the same blots as the protein of interest. Again, we believe that these proteins should have been measured in response to insulin stimulation. Another point to consider is that IR may be the result of changes at any stage of the insulin signaling pathway, so while some components of the pathway may be preserved, changes may be present in other proteins. In the study by Kim et al. (1999), for example, it was observed that individuals with T2D have IRS-1 and PI3K impairments, but not Akt (Kim et al., 1999). Moreover, we have not used the hyperinsulinemic euglycemic clamp technique (known to be the “gold standard”) for the measurement of insulin sensitivity. Using HOMA may have limited the findings of the present study. The insulin sensitivity is also influenced by sex hormones. We were not able to perform the blood measurements and muscle biopsy at the same phase of the menstrual cycle. It has been reported changes around 15% in insulin sensitivity between mid-follicular and luteal phase (Sheu, 2011). Finally, we intended to comprehensively investigate the effect of HIIT on several parameters associated with IR in obese individuals, but we were not able to investigate the presence of excess saturated free fatty acids and ceramides.

## Conclusion

In summary, our data demonstrate that an 8-week HIIT program resulted in lower IR in individuals with obesity and IR. HIIT also promoted greater expression of proteins related to oxidative metabolism in insulin resistant obese individuals. Thus, HIIT appears to be an effective exercise training stimulus for ameliorating IR associated with obesity, which may be related to increased mitochondrial protein content in skeletal muscle.

## Author Contributions

MM, FM, ER-V, and FA contributed to the study design and acquirement of ethical approval. MM, DV, KP, and JL contributed to data collection. MM, MD-P, JL, FM, JL, ER-V, and FA analyzed the data and drafted the initial manuscript. The remaining authors critically revised the manuscript. FA and ER-V are guarantors of the manuscript and take full responsibility for the work as a whole, including the study design, access to data, and the decision to submit and publish the manuscript. All authors approved the final version of the manuscript.

## Conflict of Interest Statement

The authors declare that the research was conducted in the absence of any commercial or financial relationships that could be construed as a potential conflict of interest.

## Footnotes

1 http://www.ensaiosclinicos.gov.br/rg/RBR-6g3ngr/

## References

[B1] Abdul-Ghani M. A. Matsuda M. Balas B. Defronzo R. A. (2007). Muscle and liver insulin resistance indexes derived from the oral glucose tolerance test. *Diabet. Care* 30 89–94. 10.2337/dc06-1519 17192339

[B2] Aguer C. Mccoin C. S. Knotts T. A. Thrush A. B. Ono-Moore K. Mcpherson R. (2015). Acylcarnitines: potential implications for skeletal muscle insulin resistance. *FASEB J.* 29 336–345. 10.1096/fj.14-255901 25342132 PMC4285541

[B3] Aldiss P. Betts J. Sale C. Pope M. Budge H. Symonds M. E. (2018). Exercise-induced ’browning’ of adipose tissues. *Metabolism* 81 63–70. 10.1016/j.metabol.2017.11.009 29155135 PMC5893183

[B4] Antoun G. Mcmurray F. Thrush A. B. Patten D. A. Peixoto A. C. Slack R. S. (2015). Impaired mitochondrial oxidative phosphorylation and supercomplex assembly in rectus abdominis muscle of diabetic obese individuals. *Diabetologia* 58 2861–2866. 10.1007/s00125-015-3772-8 26404066

[B5] Arad A. D. Dimenna F. J. Thomas N. Tamis-Holland J. Weil R. Geliebter A. (2015). High-intensity interval training without weight loss improves exercise but not basal or insulin-induced metabolism in overweight/obese African American women. *J. Appl. Physiol.* 119 352–362. 10.1152/japplphysiol.00306.2015 26112241

[B6] Bergstrom J. Hultman E. (1966). Muscle glycogen synthesis after exercise: an enhancing factor localized to the muscle cells in man. *Nature* 210 309–310. 10.1038/210309a0 5954569

[B7] Bird S. R. Hawley J. A. (2016). Update on the effects of physical activity on insulin sensitivity in humans. *BMJ Open Sport Exerc. Med.* 2:e000143. 10.1136/bmjsem-2016-000143 28879026 PMC5569266

[B8] Boyd J. C. Simpson C. A. Jung M. E. Gurd B. J. (2013). Reducing the intensity and volume of interval training diminishes cardiovascular adaptation but not mitochondrial biogenesis in overweight/obese men. *PLoS One* 8:e68091. 10.1371/journal.pone.0068091 23861854 PMC3702554

[B9] Bruce C. R. Anderson M. J. Carey A. L. Newman D. G. Bonen A. Kriketos A. D. (2003). Muscle oxidative capacity is a better predictor of insulin sensitivity than lipid status. *J. Clin. Endocrinol. Metab.* 88 5444–5451. 10.1210/jc.2003-030791 14602787

[B10] Burgomaster K. A. Howarth K. R. Phillips S. M. Rakobowchuk M. Macdonald M. J. Mcgee S. L. (2008). Similar metabolic adaptations during exercise after low volume sprint interval and traditional endurance training in humans. *J. Physiol.* 586 151–160. 10.1113/jphysiol.2007.142109 17991697 PMC2375551

[B11] Canto C. Auwerx J. (2009). PGC-1alpha, SIRT1 and AMPK, an energy sensing network that controls energy expenditure. *Curr. Opin. Lipidol.* 20 98–105. 10.1097/MOL.0b013e328328d0a4 19276888 PMC3627054

[B12] Carlson C. J. Rondinone C. M. (2005). Pharmacological inhibition of p38 MAP kinase results in improved glucose uptake in insulin-resistant 3T3-L1 adipocytes. *Metabolism* 54 895–901. 10.1016/j.metabol.2005.02.003 15988698

[B13] Cuenda A. Rousseau S. (2007). p38 MAP-kinases pathway regulation, function and role in human diseases. *Biochim. Biophys. Acta* 1773 1358–1375. 10.1016/j.bbamcr.2007.03.010 17481747

[B14] de Alvaro C. Teruel T. Hernandez R. Lorenzo M. (2004). Tumor necrosis factor alpha produces insulin resistance in skeletal muscle by activation of inhibitor kappaB kinase in a p38 MAPK-dependent manner. *J. Biol. Chem.* 279 17070–17078. 10.1074/jbc.M312021200 14764603

[B15] de Matos M. A. Ottone Vde O. Duarte T. C. Sampaio P. F. Costa K. B. Fonseca C. A. (2014). Exercise reduces cellular stress related to skeletal muscle insulin resistance. *Cell Stress Chaperones* 19 263–270. 10.1007/s12192-013-0453-8 23975543 PMC3933613

[B16] DeFronzo R. A. Jacot E. Jequier E. Maeder E. Wahren J. Felber J. P. (1981). The effect of insulin on the disposal of intravenous glucose. Results from indirect calorimetry and hepatic and femoral venous catheterization. *Diabetes* 30 1000–1007. 10.2337/diab.30.12.1000 7030826

[B17] Di Meo S. Iossa S. Venditti P. (2017). Skeletal muscle insulin resistance: role of mitochondria and other ROS sources. *J. Endocrinol.* 233 R15–R42. 10.1530/JOE-16-0598 28232636

[B18] Felber J. P. Golay A. (2002). Pathways from obesity to diabetes. *Int. J. Obes. Relat. Metab. Disord.* 26(Suppl. 2), S39–S45. 10.1038/sj.ijo.0802126 12174327

[B19] Fujishiro M. Gotoh Y. Katagiri H. Sakoda H. Ogihara T. Anai M. (2003). Three mitogen-activated protein kinases inhibit insulin signaling by different mechanisms in 3T3-L1 adipocytes. *Mol. Endocrinol.* 17 487–497. 10.1210/me.2002-0131 12554784

[B20] Gehart H. Kumpf S. Ittner A. Ricci R. (2010). MAPK signalling in cellular metabolism: stress or wellness? *EMBO Rep.* 11 834–840. 10.1038/embor.2010.160 20930846 PMC2966959

[B21] Gibala M. J. (2018). Interval training for cardiometabolic health: why such a HIIT? *Curr. Sports Med. Rep.* 17 148–150. 10.1249/JSR.0000000000000483 29738319

[B22] Gibala M. J. Gillen J. B. Percival M. E. (2014). Physiological and health-related adaptations to low-volume interval training: influences of nutrition and sex. *Sports Med.* 44(Suppl. 2), S127–S137. 10.1007/s40279-014-0259-6 25355187 PMC4213388

[B23] Gibala M. J. Mcgee S. L. Garnham A. P. Howlett K. F. Snow R. J. Hargreaves M. (2009). Brief intense interval exercise activates AMPK and p38 MAPK signaling and increases the expression of PGC-1alpha in human skeletal muscle. *J. Appl. Physiol.* 106 929–934. 10.1152/japplphysiol.90880.2008 19112161

[B24] Granata C. Oliveira R. S. Little J. P. Renner K. Bishop D. J. (2016) Training intensity modulates changes in PGC-1α and p53 protein content and mitochondrial respiration, but not markers of mitochondrial content in human skeletal muscle. *FASEB J.* 30 959–970. 10.1096/fj.15-276907 26572168

[B25] Hafizi Abu Bakar M. Kian Kai C. Wan Hassan W. N. Sarmidi M. R. Yaakob H. Zaman Huri H. (2015). Mitochondrial dysfunction as a central event for mechanisms underlying insulin resistance: the roles of long chain fatty acids. *Diabet. Metab. Res. Rev.* 31 453–475. 10.1002/dmrr.2601 25139820

[B26] Hansen P. A. Nolte L. A. Chen M. M. Holloszy J. O. (1998). Increased GLUT-4 translocation mediates enhanced insulin sensitivity of muscle glucose transport after exercise. *J. Appl. Physiol.* 85 1218–1222. 10.1152/jappl.1998.85.4.1218 9760308

[B27] Heilbronn L. K. Gan S. K. Turner N. Campbell L. V. Chisholm D. J. (2007). Markers of mitochondrial biogenesis and metabolism are lower in overweight and obese insulin-resistant subjects. *J. Clin. Endocrinol. Metab.* 92 1467–1473. 10.1210/jc.2006-2210 17244782

[B28] Helgerud J. Hoydal K. Wang E. Karlsen T. Berg P. Bjerkaas M. (2007). Aerobic high-intensity intervals improve VO2max more than moderate training. *Med. Sci. Sports Exerc.* 39 665–671. 10.1249/mss.0b013e3180304570 17414804

[B29] Henriksen E. J. (2002). Invited review: effects of acute exercise and exercise training on insulin resistance. *J. Appl. Physiol.* 93 788–796. 10.1152/japplphysiol.01219.2001 12133893

[B30] Hirosumi J. Tuncman G. Chang L. Gorgun C. Z. Uysal K. T. Maeda K. (2002). A central role for JNK in obesity and insulin resistance. *Nature* 420 333–336. 10.1038/nature01137 12447443

[B31] Holloway G. P. Bonen A. Spriet L. L. (2009). Regulation of skeletal muscle mitochondrial fatty acid metabolism in lean and obese individuals. *Am. J. Clin. Nutr.* 89 455S–462S. 10.3945/ajcn.2008.26717B 19056573

[B32] Holloway G. P. Perry C. G. Thrush A. B. Heigenhauser G. J. Dyck D. J. Bonen A. (2008). PGC-1alpha’s relationship with skeletal muscle palmitate oxidation is not present with obesity despite maintained PGC-1alpha and PGC-1beta protein. *Am. J. Physiol. Endocrinol. Metab.* 294 E1060–E1069. 10.1152/ajpendo.00726.2007 18349111

[B33] Hood M. S. Little J. P. Tarnopolsky M. A. Myslik F. Gibala M. J. (2011). Low-volume interval training improves muscle oxidative capacity in sedentary adults. *Med. Sci. Sports Exerc.* 43 1849–1856. 10.1249/MSS.0b013e3182199834 21448086

[B34] Hossain P. Kawar B. El Nahas M. (2007). Obesity and diabetes in the developing world–a growing challenge. *N. Engl. J. Med.* 356 213–215. 10.1056/NEJMp068177 17229948

[B35] Hwang S. L. Jeong Y. T. Li X. Kim Y. D. Lu Y. Chang Y. C. (2013). Inhibitory cross-talk between the AMPK and ERK pathways mediates endoplasmic reticulum stress-induced insulin resistance in skeletal muscle. *Br. J. Pharmacol.* 169 69–81. 10.1111/bph.12124 23373714 PMC3632239

[B36] Itani S. I. Ruderman N. B. Schmieder F. Boden G. (2002). Lipid-induced insulin resistance in human muscle is associated with changes in diacylglycerol, protein kinase C, and IkappaB-alpha. *Diabetes Metab. Res. Rev.* 51 2005–2011. 12086926 10.2337/diabetes.51.7.2005

[B37] Jager S. Handschin C. St-Pierre J. Spiegelman B. M. (2007). AMP-activated protein kinase (AMPK) action in skeletal muscle via direct phosphorylation of PGC-1alpha. *Proc. Natl. Acad. Sci. U.S.A.* 104 12017–12022. 10.1073/pnas.0705070104 17609368 PMC1924552

[B38] Jelleyman C. Yates T. O’donovan G. Gray L. J. King J. A. Khunti K. (2015). The effects of high-intensity interval training on glucose regulation and insulin resistance: a meta-analysis. *Obes. Rev.* 16 942–961. 10.1111/obr.12317 26481101

[B39] Jheng H. F. Tsai P. J. Guo S. M. Kuo L. H. Chang C. S. Su I. J. (2012). Mitochondrial fission contributes to mitochondrial dysfunction and insulin resistance in skeletal muscle. *Mol. Cell. Biol.* 32 309–319. 10.1128/MCB.05603-11 22083962 PMC3255771

[B40] Karstoft K. Winding K. Knudsen S. H. James N. G. Scheel M. M. Olesen J. (2014). Mechanisms behind the superior effects of interval vs continuous training on glycaemic control in individuals with type 2 diabetes: a randomised controlled trial. *Diabetologia* 57 2081–2093. 10.1007/s00125-014-3334-5 25099941

[B41] Keshel T. E. Coker R. H. (2015). Exercise training and insulin resistance: a current review. *J. Obes. Weight Loss Ther.* 5:S5-003. 10.4172/2165-7904.S5-003 26523243 PMC4625541

[B42] Kim Y. B. Nikoulina S. E. Ciaraldi T. P. Henry R. R. Kahn B. B. (1999). Normal insulin-dependent activation of Akt/protein kinase B, with diminished activation of phosphoinositide 3-kinase, in muscle in type 2 diabetes. *J. Clin. Invest.* 104 733–741. 10.1172/JCI6928 10491408 PMC408433

[B43] Kong Z. Fan X. Sun S. Song L. Shi Q. Nie J. (2016). Comparison of high-intensity interval training and moderate-to-vigorous continuous training for cardiometabolic health and exercise enjoyment in obese young women: a randomized controlled trial. *PLoS One* 11:e0158589. 10.1371/journal.pone.0158589 27368057 PMC4930190

[B44] Korkiakangas E. E. Alahuhta M. A. Laitinen J. H. (2009). Barriers to regular exercise among adults at high risk or diagnosed with type 2 diabetes: a systematic review. *Health Promot. Int.* 24 416–427. 10.1093/heapro/dap031 19793763

[B45] Kramer H. F. Witczak C. A. Taylor E. B. Fujii N. Hirshman M. F. Goodyear L. J. (2006). AS160 regulates insulin- and contraction-stimulated glucose uptake in mouse skeletal muscle. *J. Biol. Chem.* 281 31478–31485. 10.1074/jbc.M605461200 16935857

[B46] Little J. P. Gillen J. B. Percival M. E. Safdar A. Tarnopolsky M. A. Punthakee Z. (2011a). Low-volume high-intensity interval training reduces hyperglycemia and increases muscle mitochondrial capacity in patients with type 2 diabetes. *J. Appl. Physiol.* 111 1554–1560. 10.1152/japplphysiol.00921.2011 21868679

[B47] Little J. P. Safdar A. Bishop D. Tarnopolsky M. A. Gibala M. J. (2011b). An acute bout of high-intensity interval training increases the nuclear abundance of PGC-1alpha and activates mitochondrial biogenesis in human skeletal muscle. *Am. J. Physiol. Regul. Integr. Comp. Physiol.* 300 R1303–R1310. 10.1152/ajpregu.00538.2010 21451146

[B48] Little J. P. Safdar A. Wilkin G. P. Tarnopolsky M. A. Gibala M. J. (2010). A practical model of low-volume high-intensity interval training induces mitochondrial biogenesis in human skeletal muscle: potential mechanisms. *J. Physiol.* 588 1011–1022. 10.1113/jphysiol.2009.181743 20100740 PMC2849965

[B49] Marcinko K. Sikkema S. R. Samaan M. C. Kemp B. E. Fullerton M. D. Steinberg G. R. (2015). High intensity interval training improves liver and adipose tissue insulin sensitivity. *Mol. Metab.* 4 903–915. 10.1016/j.molmet.2015.09.006 26909307 PMC4731736

[B50] Matthews D. R. Hosker J. P. Rudenski A. S. Naylor B. A. Treacher D. F. Turner R. C. (1985). Homeostasis model assessment: insulin resistance and beta-cell function from fasting plasma glucose and insulin concentrations in man. *Diabetologia* 28 412–419. 10.1007/BF00280883 3899825

[B51] Muoio D. M. Neufer P. D. (2012). Lipid-induced mitochondrial stress and insulin action in muscle. *Cell Metab.* 15 595–605. 10.1016/j.cmet.2012.04.010 22560212 PMC3348508

[B52] Myers J. Bellin D. (2000). Ramp exercise protocols for clinical and cardiopulmonary exercise testing. *Sports Med.* 30 23–29. 10.2165/00007256-200030010-00003 10907755

[B53] Oberbach A. Bossenz Y. Lehmann S. Niebauer J. Adams V. Paschke R. (2006). Altered fiber distribution and fiber-specific glycolytic and oxidative enzyme activity in skeletal muscle of patients with type 2 diabetes. *Diabetes Care* 29 895–900. 10.2337/diacare.29.04.06.dc05-154 16567834

[B54] Sakamoto K. Holman G. D. (2008). Emerging role for AS160/TBC1D4 and TBC1D1 in the regulation of GLUT4 traffic. *Am. J. Physiol. Endocrinol. Metab.* 295 E29–E37. 10.1152/ajpendo.90331.2008 18477703 PMC2493596

[B55] Sano H. Kane S. Sano E. Miinea C. P. Asara J. M. Lane W. S. (2003). Insulin-stimulated phosphorylation of a Rab GTPase-activating protein regulates GLUT4 translocation. *J. Biol. Chem.* 278 14599–14602. 10.1074/jbc.C300063200 12637568

[B56] Sarafidis P. A. Lasaridis A. N. Nilsson P. M. Pikilidou M. I. Stafilas P. C. Kanaki A. (2007). Validity and reproducibility of HOMA-IR, 1/HOMA-IR, QUICKI and McAuley’s indices in patients with hypertension and type II diabetes. *J. Hum. Hypertens.* 21 709–716. 10.1038/sj.jhh.1002201 17443211

[B57] Schmitz-Peiffer C. Browne C. L. Oakes N. D. Watkinson A. Chisholm D. J. Kraegen E. W. (1997). Alterations in the expression and cellular localization of protein kinase C isozymes epsilon and theta are associated with insulin resistance in skeletal muscle of the high-fat-fed rat. *Diabetes Metab. Res. Rev.* 46 169–178. 9000691 10.2337/diab.46.2.169

[B58] Sethi J. K. Vidal-Puig A. J. (2007). Thematic review series: adipocyte biology. Adipose tissue function and plasticity orchestrate nutritional adaptation. *J. Lipid Res.* 48 1253–1262. 10.1194/jlr.R700005-JLR200 17374880 PMC4303760

[B59] Shaw J. E. Sicree R. A. Zimmet P. Z. (2010). Global estimates of the prevalence of diabetes for 2010 and 2030. *Diabetes. Res. Clin. Pract.* 87 4–14. 10.1016/j.diabres.2009.10.007 19896746

[B60] Sherwood N. E. Jeffery R. W. (2000). The behavioral determinants of exercise: implications for physical activity interventions. *Annu. Rev. Nutr.* 20 21–44. 10.1146/annurev.nutr.20.1.21 10940325

[B61] Sheu W. H. (2011). Alteration of insulin sensitivity by sex hormones during the menstrual cycle. *J. Diabetes Investig.* 2 258–259. 10.1111/j.2040-1124.2011.00116.x 24843494 PMC4014963

[B62] Simoneau J. A. Kelley D. E. (1997). Altered glycolytic and oxidative capacities of skeletal muscle contribute to insulin resistance in NIDDM. *J. Appl. Physiol.* 83 166–171. 10.1152/jappl.1997.83.1.166 9216960

[B63] St-Pierre J. Lin J. Krauss S. Tarr P. T. Yang R. Newgard C. B. (2003). Bioenergetic analysis of peroxisome proliferator-activated receptor gamma coactivators 1alpha and 1beta (PGC-1alpha and PGC-1beta) in muscle cells. *J. Biol. Chem.* 278 26597–26603. 10.1074/jbc.M301850200 12734177

[B64] Thompson P. D. Arena R. Riebe D. Pescatello L. S. American College of Sports Medicine (2013). ACSM’s new preparticipation health screening recommendations from ACSM’s guidelines for exercise testing and prescription, ninth edition. *Curr. Sports Med. Rep.* 12 215–217. 10.1249/JSR.0b013e31829a68cf 23851406

[B65] Vind B. F. Pehmller C. Treebak J. T. Birk J. B. Hey-Mogensen M. Beck-Nielsen H. (2011). Impaired insulin-induced site-specific phosphorylation of TBC1 domain family, member 4 (TBC1D4) in skeletal muscle of type 2 diabetes patients is restored by endurance exercise-training. *Diabetologia* 54 157–167. 10.1007/s00125-010-1924-4 20938636

[B66] WHO (2017). *Obesity and Overweight.* Available: http://www.who.int/news-room/fact-sheets/detail/obesity-and-overweight

[B67] Whyte L. J. Gill J. M. Cathcart A. J. (2010). Effect of 2 weeks of sprint interval training on health-related outcomes in sedentary overweight/obese men. *Metabolism* 59 1421–1428. 10.1016/j.metabol.2010.01.002 20153487

[B68] Willett W. Stampfer M. J. (1986). Total energy intake: implications for epidemiologic analyses. *Am. J. Epidemiol.* 124 17–27. 10.1093/oxfordjournals.aje.a114366 3521261

[B69] Wright D. C. Han D. H. Garcia-Roves P. M. Geiger P. C. Jones T. E. Holloszy J. O. (2007). Exercise-induced mitochondrial biogenesis begins before the increase in muscle PGC-1alpha expression. *J. Biol. Chem.* 282 194–199. 10.1074/jbc.M606116200 17099248

[B70] Wu H. Ballantyne C. M. (2017). Skeletal muscle inflammation and insulin resistance in obesity. *J. Clin. Invest.* 127 43–54. 10.1172/JCI88880 28045398 PMC5199705

[B71] Wu Z. Puigserver P. Andersson U. Zhang C. Adelmant G. Mootha V. (1999). Mechanisms controlling mitochondrial biogenesis and respiration through the thermogenic coactivator PGC-1. *Cell* 98 115–124. 10.1016/S0092-8674(00)80611-X 10412986

[B72] Zhang L. Feng Y. List J. Kasichayanula S. Pfister M. (2010). Dapagliflozin treatment in patients with different stages of type 2 diabetes mellitus: effects on glycaemic control and body weight. *Diabetes. Obes. Metab.* 12 510–516. 10.1111/j.1463-1326.2010.01216.x 20518806

[B73] Zhang Y. Uguccioni G. Ljubicic V. Irrcher I. Iqbal S. Singh K. (2014). Multiple signaling pathways regulate contractile activity-mediated PGC-1alpha gene expression and activity in skeletal muscle cells. *Physiol. Rep.* 2:e12008. 10.14814/phy2.12008 24843073 PMC4098736

[B74] Zierath J. R. Krook A. Wallberg-Henriksson H. (2000). Insulin action and insulin resistance in human skeletal muscle. *Diabetologia* 43 821–835. 10.1007/s001250051457 10952453

